# The Rational Treatment of Gastric and Intestinal Disorders

**Published:** 1898-06

**Authors:** 


THE RATIONAL TREATMENT OF GASTRIC AND INTESTINAL DISORDERS. By Charles Marchand. Second edition. New York. 28 Prince Street. 1898.

This little pamphlet contains abstracts of scientific articles by various contributors to medical literature.

It contains such articles as rational treatment of gastric and intestinal disorders, with a statement of the proper requirements of an antiseptic. These requirements are fully met by hydrozone, which has been mentioned in these pages before, and the advertisement of which may be found in this number.

Its value in the treatment of yellow fever, typhoid fever, cholera infantum, and Asiatic cholera is fully set forth.

Its use in the treatment of chronic gastric catarrh is fully given, and its use, even in treatment of insanity is given. According to this article, "there is no more prolific source of insanity than auto-infection."

To meet this condition intestinal antisepsis must be rigidly enforced, and for the accomplishment of it, hydrozone, in a three per cent. solution, must be administered.

Chronic gastritis and chronic catarrh of the stomach are treated successfully by the use of hydrozone, and the same is true of the entero-colitis of infancy.

Those who are interested may secure a copy of this little work by writing to the author, Mr. Charles Marchand, 28 Prince Street, New York.

